# Melanoma Management: Exploring Staging, Prognosis, and Treatment Innovations

**DOI:** 10.3390/ijms25115794

**Published:** 2024-05-26

**Authors:** Walid Shalata, Zoe Gabrielle Attal, Adam Solomon, Sondos Shalata, Omar Abu Saleh, Lena Tourkey, Fahed Abu Salamah, Ibrahim Alatawneh, Alexander Yakobson

**Affiliations:** 1 The Legacy Heritage Cancer Center and Larry Norton Institute, Soroka Medical Center, Beer Sheva 84105, Israel; 2 Faculty of Health Sciences, Ben-Gurion University of the Negev, Beer Sheva 84105, Israel; 3 Medical School for International Health, Ben-Gurion University of the Negev, Beer Sheva 84105, Israel; 4 Nutrition Unit, Galilee Medical Center, Nahariya 22000, Israel; 5 Department of Dermatology and Venereology, The Emek Medical Centre, Afula 18341, Israel; 6 Department of Dermatology, Soroka Medical Center, Beer Sheva 84105, Israel

**Keywords:** melanoma, metastatic, immunotherapy, tyrosine kinase inhibitors, BRAF, skin cancer, brain metastasis

## Abstract

Melanoma, a malignant neoplasm originating from melanocytes, stands as one of the most prevalent cancers globally, ranking fifth in terms of estimated new cases in recent years. Its aggressive nature and propensity for metastasis pose significant challenges in oncology. Recent advancements have led to a notable shift towards targeted therapies, driven by a deeper understanding of cutaneous tumor pathogenesis. Immunotherapy and tyrosine kinase inhibitors have emerged as promising strategies, demonstrating the potential to improve clinical outcomes across all disease stages, including neoadjuvant, adjuvant, and metastatic settings. Notably, there has been a groundbreaking development in the treatment of brain metastasis, historically associated with poor prognosis in oncology but showcasing impressive results in melanoma patients. This review article provides a comprehensive synthesis of the most recent knowledge on staging and prognostic factors while highlighting emerging therapeutic modalities, with a particular focus on neoadjuvant and adjuvant strategies, notably immunotherapy and targeted therapies, including the ongoing trials.

## 1. Introduction

Cutaneous melanoma originates from melanocytes in the skin. It is commonly associated with ultraviolet radiation exposure from natural sunlight and indoor tanning, although there exist subtypes of predisposing factors unrelated to exposure, such as family history, and number of nevi, older age, and male sex. Primary melanomas typically exhibit dark pigmentation, although some can be amelanotic or hypomelanotic. While clinical assessment raises suspicion of melanoma, a histopathological assessment is necessary to confirm diagnosis [1,2,3,4].

Malignant melanoma, one of the most prevalent cancers globally, ranked 20% in terms of estimated new cases according to Cancer Statistics Cases (21.2 new cases per 100,000 men and women each year) [4,5,6]. While comprising only 4–7% of skin cancers, melanoma is responsible for the majority of skin cancer fatalities [4]. Specifically, melanoma of the skin has shown a yearly increase in incidence and is projected to account for over 100,000 new cases and over than 8000 deaths in the USA in 2023 alone, especially within white populations [7]. Approximately 4% of all annually diagnosed patients will eventually progress to stage 4 metastatic melanoma [8,9].

While surgery is the primary treatment modality in early-stage disease, treatment options were historically limited for advanced stages. However, there has been a significant shift in the treatment landscape for advanced melanoma with the advent of immunotherapy and targeted therapies. This transformation began in 2011 with the U.S. Food and Drug Administration (FDA) approval of Ipilimumab, the first drug demonstrated to reduce mortality rates. Since then, the FDA has sanctioned several additional immune checkpoint inhibitors (ICIs) for melanoma treatment, resulting in improved overall survival rates [9,10,11]. 

The BRAF gene is responsible for encoding a protein kinase (MAPK) crucial for regulating cellular growth and proliferation within tumor cells. Mutations in the BRAF can lead to the continuous activation of the MAPK pathway. The predominant mutation detected in melanoma patients involves substituting a valine with glutamic acid at the amino acid position 600 (V600E). About 40–60% of melanomas exhibit activating BRAF mutations, with V600E mutations accounting for 70–90% of these cases [2,3,4,8,9,10].

The analysis of BRAF mutation status has become a standard diagnostic procedure for melanoma. While most patients exhibit homogeneous BRAF mutations, heterogeneity has been observed, although its impact on therapeutic response to BRAF/MEK inhibitors or patient survival remains under investigation. Notably, combining BRAF and MEK inhibitors has demonstrated even greater efficacy. Additionally, the association between BRAF mutation and rapid disease progression has led to the development of BRAF inhibitors, representing a significant advancement in melanoma treatment. These inhibitors have shown efficacy both as monotherapy in patients with unresectable, metastatic melanoma and as adjuvant therapy in stage III melanoma patients with BRAF mutations [12,13,14].

In first-line therapy, the 3-year overall survival (OS) rates averaged 41.3% for BRAF plus MEK inhibitor, and the 5-year OS rates averaged 34%; for PD-1 inhibition, the 3-year OS rates were 58.4%, and the 5-year OS rates were 44%; and for CTLA-4 plus PD-1 inhibition, the 5-year OS rates was 52% [8,9].

In the following review, we incorporated the latest insights into staging and prognostic factors, emphasizing emerging therapeutic approaches. Our focus lies on neoadjuvant and adjuvant strategies, particularly highlighting immunotherapy and targeted therapies, including ongoing clinical trials.

## 2. Materials and Methods

Numerous searches were conducted in PubMed and ClinicalTrials.gov, spanning from their inception to March 2024, aiming to identify clinical trials encompassing neoadjuvant, adjuvant, and metastatic treatments involving ICI’s, BRAF, and MEK inhibitors in melanoma. Search terms employed comprised “melanoma”, “melanoma stages”, “melanoma diagnosis”, “melanoma treatment”, “early-stage melanoma”, “locally advanced melanoma”, “metastatic melanoma”, “neoadjuvant BRAF and MEK”, “neoadjuvant immunotherapy”, “adjuvant BRAF and MEK”, and “adjuvant immunotherapy”, “ipilimumab plus nivolumab in melanoma” and “pembrolizumab in melanoma”. Our review encompasses all completed trials for which results were available. The selection of ongoing trials was based on their phase and the treatment administered. All ongoing phase 1, 2, and 3 trials were included. Additionally, ongoing trials assessing novel ICIs were incorporated into the review.

## 3. Staging and Prognostic Factors

### 3.1. Staging for Melanoma

In 2018, the World Health Organization (WHO) published an updated classification of melanoma that included an array of factors, including genetics, clinical diagnosis, biomarkers, and epidemiology [15]. In previous studies examining the relationship between sun exposure and BRAF mutation, researchers divided skin into sun-exposed and sun-shielded categories, revealing that BRAF mutations are more prevalent in young individuals with sporadic sun exposure than in those chronically exposed to sunlight. These findings indicate that BRAF-mutated lesions often appear during the early stages of life with limited UV exposure. This is in contrast to BRAF wild-type melanomas, which usually develop after prolonged exposure to high doses of UV radiation, leading to solar elastosis [8,16,17].

This classification by the WHO considers the gold standard prognostic markers (Clark level ulceration, mitotic rate, and Breslow thickness), describes the original tumor, and establishes a prognosis. In addition to that, clinical evaluation of the melanoma is necessary to determine via which of the two above-mentioned pathways it developed from. Sun exposure tends to be associated with mutations such as BRAF and NRAS [8]. If neither of these mutations is found, oncogenes such as CDK4 can also be associated with sun exposure. 

### 3.2. Prognostic Factors

In contrast to many other cancers, the prognosis of melanoma is not influenced by the tumor’s molecular profile. Instead, the primary determinant of prognosis in malignant melanoma is the Breslow thickness. Other crucial prognostic factors routinely assessed during the pathological examination encompass ulceration, mitosis rate, surgical margins, and Clark level, although recently, the significance of Clark level has diminished.

#### 3.2.1. LDH

High levels of lactate dehydrogenase (LDH) stand out as a significant independent prognostic factor in metastatic melanoma. LDH leakage into the serum is believed to occur when melanoma cells outgrow their blood supply. The escalation of LDH levels emerges as an adverse prognostic marker, irrespective of metastasis site or number, strongly correlating with reduced survival rates in advanced disease [18,19]. The American Joint Committee on Cancer’s staging system uses elevated LDH levels alongside any distant metastasis to classify patients into stage IV [18]. Moreover, elevated serum LDH levels act as a negative predictor for therapy response. A recent randomized study exploring the efficacy of dacarbazine with and without oblimersen in advanced melanoma patients found that supplementing with oblimersen resulted in improved overall response rates and survival. Notably, LDH emerged as a highly predictive factor for the effects of oblimersen [18,19,20,21,22,23]. This discovery bears significance for clinical trials, emphasizing the importance of stratifying patients based on LDH levels to prevent high baseline LDH levels from masking treatment effects [18,19,20,21,22,23].

#### 3.2.2. Plasma Membrane Calcium-Transporting ATPase 4 (PMCA4)

PMCA4 is an enzyme encoded by the AATP2B4 gene in humans. PMCA4 plays an important role in maintaining intracellular calcium balance by expelling bivalent calcium ions from eukaryotic cells against substantial concentration gradients. PMCA4 is implicated in various cancer types, including pancreatic, breast, and colon cancer [24,25,26,27]. In the context of melanoma, preclinical studies indicate that PMCA4, a plasma membrane calcium ATPase, inhibits the migration and metastatic potential of BRAF-mutant melanoma cells. Consequently, downregulation of PMCA4 may contribute to heightened melanoma cell migration and metastasis [25,26,27,28,29,30].

A recent study investigated the prognostic significance of PMCA4 mRNA levels in cutaneous melanoma, both in non-metastatic stages (stages I–III) and following PD-1 blockade in advanced disease. Results demonstrated that patients exhibiting high PMCA4 transcript levels experienced significantly prolonged progression-free survival. Furthermore, elevated transcript levels, as determined via RNA-seq analysis of cutaneous melanoma, were associated with extended OS and improved prognosis following PD-1 blockade [31,32,33].

### 3.3. Genomic Subtypes

Melanomas can exhibit different genomic subtypes that influence the course of disease and survival. Neurofibromin 1, NRAS, KIT, and BRAF represent the majority of cases and are thus outlined below. 

#### 3.3.1. Neurofibromin 1 (NF1)

The NF1 gene encodes neurofibromin, a key negative regulator of the RAS protein via its GTPase activity. As a type of tumor suppressor gene, NF1 mutations are associated with a common genetic syndrome characterized by café-au-lait macules, neurofibromas, and other manifestations. Individuals with NF1 mutations face an elevated risk of developing desmoplastic melanoma, which accounts for 10–15% of melanomas. NF1-mutated melanomas typically arise on chronically sun-exposed skin or in older individuals, exhibit a high mutation burden, and lack BRAF or NRAS mutations [34,35,36].

#### 3.3.2. NRAS

The NRAS gene is responsible for encoding the NRAS protein, primarily involved in regulating cell division. NRAS is a proto-oncogene, and mutations in this gene can contribute to cancer development in humans. NRAS mutations are identified in approximately 15–20% of tested melanomas. However, they do not reliably predict disease prognosis. Nevertheless, in stage 4 melanoma, NRAS mutation serves as an independent predictor, indicating shorter survival [37,38,39].

#### 3.3.3. KIT

The c-KIT gene, classified as a proto-oncogene, governs the production of transmembrane receptor tyrosine kinase. KIT signaling plays a pivotal role in cell survival, proliferation, and differentiation. While KIT mutations are detected in only 3% of melanomas overall, they are notably prevalent and found in approximately 35% of acral and mucosal melanomas. Contrary to BRAF and NRAS mutations, KIT mutations seldom coincide with them, leading to the exploration of therapeutic avenues involving KIT inhibitors. Unlike NRAS and BRAF mutations, KIT mutations do not correlate with specific histological subtypes or tumor stages. However, they exhibit close associations with advancing age, acral and mucosal subtypes of melanoma and sites of chronic sun-induced damage [40,41,42].

#### 3.3.4. BRAF

BRAF is a proto-oncogene categorized within the signal transduction protein kinases, playing a pivotal role in regulating cell proliferation, differentiation, and migration. Mutations in this gene have been identified across various cancer types, with its association with malignant melanoma extensively researched. In melanoma, the BRAF gene is the most commonly mutated, with the BRAF subtype detected in approximately 50% (40% to 60%) of individuals, activating the mitogen-activated protein (MAP) kinase pathway [43,44]. The majority of BRAF mutations occur in amino acid 600 of the gene, resulting in the discovery of the V600E, V600K, and V600R point mutations. The V600E mutation, predominant in 80% of BRAF-related melanomas, is often found in younger patients at anatomical sites shielded from sun exposure. Understanding the BRAF mutation status has become standard practice in diagnosing melanoma. However, BRAF mutations alone are typically insufficient to initiate tumor formation, as they are also commonly found in most benign nevi [4,45,46,47,48].

## 4. Neoadjuvant Therapy

Neoadjuvant immunotherapy is described as treatment administered to reduce tumor size (down-staging) prior to mainstay treatment (typically surgery), improving surgical outcomes. Neoadjuvant therapy includes many different methods of treatment, such as chemotherapy, hormone therapy, radiation, or a combination of these. Neoadjuvant’s benefits include an understanding of the tumor’s microenvironment while the patient is undergoing active treatment [49]. It can help assess the effectiveness of the treatment and guide further decisions. 

However, neoadjuvant therapy is not without its disadvantages. For instance, in conditions like stage III melanoma, there is a proportion of patients who fail screening upon enrolment in neoadjuvant studies. This is frequently due to underestimated metastases or rapid disease progression, outnumbering those who are unable to proceed to surgery. Additionally, adverse effects can be severe and irreversible, potentially causing delays in surgical intervention. 

Nonetheless, research shows that neoadjuvant therapy effectively targets immune checkpoints in melanoma, leading to promising outcomes. A summary of the past and ongoing clinical trials for neo-adjuvant therapy administered to melanoma patients are listed in Table 1.

### 4.1. Past Clinical Trials

#### 4.1.1. Neo-Adjuvant or Adjuvant-Only Pembrolizumab in Advanced Melanoma (NCT03698019)

In melanoma, administering pembrolizumab as a neoadjuvant therapy followed by adjuvant therapy showed better outcomes than patients receiving pembrolizumab as standard treatment alone. At 2 years, the neoadjuvant–adjuvant group showed 72% event-free survival (EFS) compared to 49% in the adjuvant-alone group [50]. 

#### 4.1.2. OpACIN and OpACIN-Neo

In 2023, the randomized controlled trials (RCT) named OpACIN and OpACIN-neo supported the use of neoadjuvant therapy for patients with high-risk stage III melanoma. The primary endpoints were safety (immune-related toxicity), radiological objective response, and pathological response [50]. The OpACIN-neo trial demonstrated the use of two cycles of nivolumab (NIVO) 3 mg/kg and ipilimumab (IPI) 1 mg/kg conserved high pathologic response rates (77%) while simultaneously decreasing toxicity. Thus, neoadjuvant therapy combined with IPI and NIVO has been demonstrated as safe and recommended, inducing a broader T-cell response [52]. 

#### 4.1.3. PRADO

The personalized response-directed surgery and adjuvant therapy (PRADO) extension cohort confirmed the findings of the OpACIN-neo trial [52]. Simultaneously, they proved that implementing ilioinguinal lymph node (ILN) resection in conjunction with neoadjuvant therapy proved safe and efficient. The concern of surgery delay was eliminated as only a small subset of patients experiencing immune-related adverse effects were affected. 

#### 4.1.4. Neoadjuvant Relatlimab and Nivolumab in Resectable Melanoma (NCT02519322)

The Neoadjuvant relatlimab and NIVO study investigated the use of these two neoadjuvant treatments in resectable clinical stage III or oligometastatic stage IV melanoma [53]. Patients were given two doses of NIVO 480 mg and relatlimab 160 mg intravenously (IV) every 4 weeks. This was followed by surgical management, after which they were administered ten doses of an adjuvant combination. Neoadjuvant therapy with relatlimab and NIVO resulted in a major pathologic response rate in 57% of patients. The 2-year RFS was also significantly improved in those with any pathologic response (92%) versus those with no pathologic response (55%). No grade 3 and above immune-related adverse effects (IRAEs) were noted for neoadjuvant therapy. However, 26% of grade 3 and 4 IRAEs were reported in the adjuvant context. Interestingly, a third of the patients discontinued their adjuvant therapy before completing the trial due to toxicity. The disease recurrence rate during follow-up was very low. Consequently, it remains uncertain whether the addition of adjuvant therapy is necessary to further mitigate the risk of recurrence. 

In summary, these results align with previous clinical trials, indicating that neoadjuvant therapies like relatlimab and NIVO are effective and well tolerated in treating stage III melanoma.

### 4.2. Ongoing Clinical Trials

#### The NADINA Trial

The NADINA trial is a phase 3 RCT aiming to compare the use of neoadjuvant IPI and nivolumab with standard nivolumab use in resectable stage III melanoma. This trial follows the aforementioned OpACIN, OpACIN neo, and PRADO trials. The OpACIN-neo trials showed that patients with lower pathologic response had a suboptimal prognosis, and adjuvant therapy could prove beneficial. The NADINA trial is still ongoing and will analyze 420 patients randomized to neoadjuvant or adjuvant therapy [54]. They aim to calculate EVS following treatment of stage III melanoma of patients separated into groups A and B, which were administered two different protocols. Patients will be separated by BRAF mutation, continent, and in-transit metastasis (ITM). Then, group A will receive two cycles of IPI 80 mg plus NIVO 240 mg followed by therapeutic lymph node dissection (TLND) after 6 weeks. Where BRAF^V600^ mutation is present, patients will receive adjuvants dabrafenib plus trametinib (dab plus tram) for 46 weeks. Group B will undergo TLND followed by 12 cycles of 480 mg of NIVO. 

## 5. Adjuvant Therapy

Adjuvant therapy is administered post- melanoma surgery to diminish the risk of recurrence. Such therapies may involve immunotherapy or targeted treatment. Traditionally deemed “curative”, it effectively lowers disease morbidity by thwarting recurrence. Following surgical excision of the cancer, adjuvants target any residual microscopic disease, aiming to eliminate it [55]. A summary of the past and ongoing clinical trials for adjuvant therapy administered to melanoma patients is listed in Table 2.

### 5.1. Past Clinical Trials for Stage IIB/IIC Melanoma

#### 5.1.1. KEYNOTE-716

KEYNOTE-716 was an RCT that investigated the use of pembrolizumab as an adjuvant therapy for patients with fully resected stage II melanoma in 160 academic medical centers. The estimated 18-month survival rate with no recurrence was higher in pembrolizumab (86%) than in the placebo group (77%). Overall, pembrolizumab reduced the risk of recurrence by 35%. 

Notwithstanding, 80% of patients experienced an adverse effect from all causes combined. For example, in the pembrolizumab cohort, 25% of patients experienced endocrine disorders (primarily hypothyroidism) compared to 5% in the placebo group. 

Significantly, distant metastases were less frequent (as a first recurrence of disease) in the pembrolizumab group (6%) than the placebo group (12%), which the authors of KEYNOTE-716 intend to continue following and report on. The patients’ quality of life in the pembrolizumab group was preserved throughout the trial. 

Finally, while adjuvant therapy is common for stage III melanoma, it is not for stages IIB and IIC, although 10-year melanoma-specific survival rates are comparable for stages IIC (75%), IIB (82%), and IIIB (77%) melanoma. Until data on survival are collected, it is unclear whether pembrolizumab adjuvant therapy will be used for stages IIB and IIC melanoma, although it is promising [56]. 

#### 5.1.2. CheckMate 76K

The CheckMate 76K trial followed the KEYNOTE-716 trial, and while results between clinical trials cannot be directly compared due to different study designs, they simultaneously highlight promising results for the use of adjuvant therapy in resected stages IIB/IIC melanoma [57]. In this phase 3 double-blind trial, 790 patients were either assigned to NIVO 480 mg or placebo every 4 weeks for 1 year. NIVO showed a 58% risk of recurrence reduction and/or death compared to placebo, in addition to a 53% reduction in the risk of distant metastases. This echoes the results of the KEYNOTE 716 trial. The findings of this trial demonstrate NIVO as an adequate, efficient adjuvant therapy for resected stages IIB and IIC melanoma. However, it is not deprived of adverse effects, with 10.3% of NIVO patients experiencing them versus 2.3% of placebo patients. 

### 5.2. Past Clinical Trials for Stages III and IV Resectable Melanoma

#### 5.2.1. Keynote 053 (Pembrolizumab vs. Interferon alfa-2b or Ipilimumab)

This phase 3 RCT investigated the use of pembrolizumab (for 1 year) compared to high-dose *interferon alfa-2b* (IFNα-2b) for 1 year, or IPI for up to 3 years, in resected stage III and IV melanoma [58]. The aim was to compare the recurrence-free survival rate (RFS) and OS between the different therapies. At median follow-up, pembrolizumab had better RFS when compared to comparator therapies. Such an improvement was not determined as statically significant for OS. 

Additionally, adverse events grades 3 and above were 19.5% with pembrolizumab, 71.2% with IFNα-2b, and 49.2% with IPI. For example, fatigue (59%) and maculopapular rash (29%) represented a majority of the pembrolizumab side effects, while fatigue (51%) and diarrhea (48%) were frequent in the IPI group. In the IFNα-2b patients, fatigue (88%) and aspartate aminotransferase increase (70%) were the most common side effects. 

Following this study, it is safe to suggest that pembrolizumab has a reduced RFS compared to the prior standard of care therapy. 

#### 5.2.2. EORTC 18071

The EORTC 18071 trial is a double-blind RCT assessing the use of IPI as an adjuvant treatment for patients with resected stage III melanoma at high risk of recurrence [59]. A total of 951 patients were treated with IPI or placebo to include in intention-to-treat analyses. The protocol consisted of 4 doses of IV infusions of 10 mg/kg or placebo every 3 weeks, after which they were followed every 3 months for up to 3 years. The median RFS was 9 months longer in the IPI group (26.1 months) versus placebo (17.1 months). Grade 3/4 IRAEs in the IPI-treated group were mostly gastrointestinal (16%), hepatic (11%) and endocrine (8%). In the placebo group, these respectfully represented <1%, <1%, and none. Similarly to the *NCT02519322* trial, discontinuation of treatment was quite prevalent. Over half (52%) of IPI-treated patients stopped treatment. However, we can conclude that the use of IPI adjuvant therapy improved the RFS. The IRAEs, while high, were on par with adverse effects typically observed in advanced stage IV melanoma. This suggests that the IRAE profile of IPI is not a deterrent from adjuvant therapy. The five-year overall survival rate was 65.4% in the ipilimumab group, whereas it stood at 54.4% in the placebo group (*p* = 0.001) [60]. 

#### 5.2.3. CheckMate 915

The CheckMate 915 was a randomized double-blind phase 3 trial investigating the combined use of IPI and NIVO compared to NIVO only in resected Stage IIB-D and IV melanoma [61]. A total of 1833 patients were assigned to either of the two protocols. An amount of 916 patients were given NIVO 240 mg once every 2 weeks and IPI 1 mg/kg once every 6 weeks, while 917 patients received NIVO 480 mg once every 4 weeks only. The patients were stratified by the tumor stage and programmed death ligand 1 (PDL-1) expression status. Respectively, 34.6% and 11.3% of patients discontinued treatment due to drug toxicity. In the NIVO plus IPI group, recurrence events happened in 35.5% of patients, while that number was 37.6% for NIVO only. This recurrence was mainly attributed to distant metastases in both groups (49.2% vs. 45.2%).

Overall, the combined use of NIVO plus IPI did not increase the RFS when compared to NIVO therapy in this cohort of patients. When distinguishing the PD-L1 expression, recurrence events occurred at a similar rate in both cohorts (45.6% vs. 47.3%). The median RFS, however, was 6 months longer in the combination group. 

In summary, the use of combination adjuvant therapy did not show improved RFS when compared to NIVO monotherapy in Stage IIB-D and IV melanoma or in the PD-L1 expression groups. 

#### 5.2.4. EORTC 1325-MG/KEYNOTE-054

The EORTC 1325/KEYNOTE-054 is a phase 3, double-blind trial evaluating the use of pembrolizumab compared to placebo in 1019 patients with resected high-risk stage IIIA/B/C melanoma. Patients were given pembrolizumab or placebo for 3 weeks via intravenous infusions for up to 18 doses over the course of a year. Pembrolizumab demonstrated a significant benefit in distant metastasis-free survival (DMFS) with 65.3% at 3.5 years follow-up compared to 49.4% in the placebo group [62]. These results were comparable in the PD-L1 group, and the benefits of pembrolizumab were equally prevalent in the BRAF mutation population. IRAE of grade 3 or higher were comparable in placebo and pembrolizumab, with pneumonitis (1.9% vs. 1.1%), pneumonia (1.7% vs. 1.3%) and colitis (1.7% only present in pembrolizumab), febrile neutropenia (1.1% only in the placebo group). 

In a more recent follow-up, Eggermont et al. reported a continued improvement in the long-term recurrence and DMFS for pembrolizumab-treated patients with resected stage III melanoma 5 years since the EORTC 1325/KEYNOTE-054 clinical trial [63]. Compared to the placebo control, the hazard ratio for recurrence or death was 0.61, and the 5-year DMFS was about 15% higher in the pembrolizumab group. 

#### 5.2.5. Adjuvant Nivolumab Versus Ipilimumab in Resected Stage III or IV Melanoma (CheckMate 238)

This randomized, phase 3 double-blind trial investigated the use of adjuvant therapy with NIVO compared to IPI in 906 patients with resected IIB/C or IV melanoma [64]. Patients were administered either NIVO 3 mg/kg every 2 weeks or IPI 10 mg/kg every 3 weeks for four doses and then every 12 weeks for a total of up to 1 year. The median RFS was not reached in any of the two groups. Nevertheless, the rate of RFS was 10% higher in the NIVO group (70.5% vs. 60.8%) than in IPI-treated patients. 

IRAEs, grade 3 or 4, were reported in 14.4% of patients in the NIVO group and in 45.9% in IPI. In both groups, skin disorders were common adverse effects, including dermatitis, rash, or pruritus. Gastrointestinal disorders and endocrine disorders were also prevalent in both treatment groups. Thus, adjuvant therapy with NIVO was more efficacious and safer than IPI as it resulted in longer RFS. 

#### 5.2.6. Distant Metastasis-Free Survival Results from the Randomized, Phase 2 mRNA-4157-P201/KEYNOTE-942 Trial

The mRNA-4157-P201/Keynote-942 trial is an open-label randomized phase 2 investigation of mRNA-centered personalized cancer vaccination [65]. This trial aimed to assess the RFS and DMFS in patients with completely resected stage IIB/C/D and IV melanoma. In total, 157 patients were administered either mRNA-4157-P201 alone (1 mg IM every 3 weeks for nine doses) or in combination with pembrolizumab (200 mg IV every 3 weeks, up to 18 cycles). At 18 months follow-up, RFS rates were higher in the combination group (78.6%) versus 62.2% in monotherapy. Similarly, DMFS showed significant improvement in the combination arm (HR = 0.347; 95% CI (0.145, 0.828), *p*-value 0.0063), with 18 month follow-up rates being 91.8% in the mRNA-4157-P201 and pembrolizumab patients compared to 76.8% in the monotherapy group. While the phase 3 trial has not yet yielded conclusions, this phase 2 trial demonstrates the combination adjuvant as more efficacious with increased RFS and DMFS at follow-up.

### 5.3. Clinical Trials for BRAF-Positive Melanoma

#### Five-Year Analysis of Adjuvant Dabrafenib plus Trametinib in Stage III Melanoma (COMBI-AD)

The COMBI-AD trial is a double-blind, randomized phase 3 trial conducted on 870 patients to assess the use of dab plus tram as adjuvant therapy in Stage III melanoma [66]. This included patients with BRAF V600E or V600K mutations. The patients administered the combined regimen were given 150 mg of dab twice daily and 2 mg of tram once daily for a total of 12 months. The placebo group had a similar treatment schedule. Patients receiving the combined regimen showed longer RFS at the 5-year analysis than the placebo group, with a 49% reduction in the risk of relapse or death. Similarly, the DMFS was 65% in the combined treatment group versus 54% in the placebo cohort.

IRAEs of grade 3 or 4 associated with dab plus tram were present but transient, therefore no meaningful difference was noted between the placebo and combined treated groups. In summary, the 5-year analysis demonstrates the benefits of 12 months of combined adjuvant therapy with long lasting effects well beyond the treatment timeframe. 

### 5.4. Clinical Trials for BRAF-Positive Melanoma

#### 5.4.1. COLUMBUS-AD Study Design (NCT05270044)

COLUMBUS-AD is a triple-blind, phase 3 trial investigating the use of adjuvant encorafenib plus binimetinib compared to placebo in completely resected IIB/C BRAF^V600^ mutated melanoma [67]. A total of 815 patients will be administered either combination therapy (450 mg once daily of encorafinib and binimetinib 45 mg twice daily) or matched placebo for up to 12 months. They will then be followed up every 3 months for 3 years and then regularly for 10 years to estimate RFS, DMFS, OS, safety, tolerability, and quality of life in both groups. Conclusions on the efficacy of this treatment are to follow, as this treatment can prove promising for stage II BRAF mutated melanoma.

#### 5.4.2. KEYVIBE-010-Ongoing

KEYVIBE-010 was an adjuvant trial building from the success of the EORTC 1325/KEYNOTE-054, which used prembrolizumab for resected high-risk stage IIIA/B/C melanoma by including coformulated vibostolimab with pembrolizumab in patients with high-risk stage IIB-IV melanoma [67]. For this phase 3 study, pembrolizumab was used in the control group, testing the hypothesis that the coformulation of both adjuvant immunotherapies may further improve outcomes [68]. Vibostolimab, an anti-TIGIT monoclonal antibody, showed success in the treatment of non-small-cell lung cancer when added to pembrolizumab in the KEYVIBE-001 phase 1 clinical trial [69]. 

In this trial, 1560 patients were randomly assigned to pembrolizumab alone or with the coformulated combination of 200 mg pembrolizumab and vibostolimab at a 1:1 ratio every 3 weeks for 17 cycles, unless discontinuation is warranted as a result of recurrence, toxicity, or patient withdrawal from the study. Results are yet to be published, as the trial is currently underway. 

## 6. Treatment of Metastatic Melanoma

### 6.1. TKI and IO Therapy for BRAF-Mutant Metastatic Melanoma Patients

#### 6.1.1. COMBI-d and COMBI-v

The authors of this study examined the long-term outcomes of patients with BRAF^V600E/K^ mutated metastatic melanoma treated with dab and tram in the COMBI-d and COMBI-v trials [70]. The 5-year outcomes showed a median PFS and OS of 11.1 months and 25.9 months, respectively. Factors associated with longer PFS and OS included normal lactate dehydrogenase levels and fewer metastatic organ sites. Complete response was observed in 19% of patients, for whom a 5-year OS rate of 71% was noted. Adverse events were reported in 98% of patients, with no unexpected events. Overall, dab plus tram led to long-term survival in one-third of patients, with the complete response being a strong predictor of prolonged benefit.

#### 6.1.2. coBRIM

coBRIM evaluated the efficacy and safety of cobimetinib and vemurafenib compared to vemurafenib in patients with untreated BRAF^V600^-mutant metastatic melanoma. A total of 495 eligible patients were enrolled in this phase 3 double-blind, randomized, and placebo-controlled clinical trial [71]. Median follow-up was 21.2 months for cobimetinib plus vemurafenib and 16.6 months for the control group.

Initial analysis showed significant improvements in PFS (12.3 months versus 7.2 months) for cobimetinib plus vemurafenib versus the control. An updated analysis at 18.5 months median follow-up demonstrated improved OS with cobimetinib plus vemurafenib compared to the control group (22.3 months versus 17.4 months). Extended follow-up after 5 years from the last patient randomized confirmed long-term benefits and identified prognostic factors associated with treatment outcomes [72]. Namely, favorable outcomes were observed in patients with normal LDH levels and low tumor burden, underscoring the importance of identifying prognostic factors to guide treatment decisions. As expected, patients achieving complete response had the best long-term survival outcomes.

However, while treatment-related adverse events (TRAEs) were nearly ubiquitously encountered in the study for both groups, high-grade events were more frequent with cobimetinib plus vemurafenib, with serious adverse events occurring in 42% of cobimetinib plus vemurafenib patients compared to 29% with the control. However, the safety profile remained consistent with previous reports, with no new safety signals detected.

#### 6.1.3. COLUMBUS 5-Year Update

This article describes the 5-year update of the COLUMBUS phase 3, triple-blind clinical trial investigating the use of adjuvant encorafenib plus binimetinib compared to Vemurafenib or encorefanenib in patients with BRAF^V600^ mutated melanoma [73]. The use of encorefenib and bimitenib has been proven efficient in previous clinical trials, with average OS rates of 32% and progression-free survival (PFS) close to 16%. In this clinical trial, a total of 577 patients were administered either the combination therapy (450 mg once daily of encorafinib and binimetinib 45 mg twice daily) or vemurafenib (960 mg twice daily) or encorafenib (300 mg once daily). Patients receiving the combined therapy had a median OS of 39.2 months compared to 16.9 months for vemurafenib monotherapy and 23.5 months for encorafenib. Similarly, the median RFS was better in the combined therapy group (14.9 months versus 7.3 for Vemurafenib therapy and 9.6 months for encorafenib). 

To note, however, in the combined therapy cohort, a staggering 67% of patients discontinued therapy (55% due to progression and 12% because of IRAEs). In the combined therapy cohort, Grade 3 and above IRAEs included increased alanine aminotransferase (13%), increased aspartate aminotransferase (11%), increased blood creatinine (5%), headache (4%), and rash (2%). Similarly, in vemurafenib and encorafenib monotherapies, the side effects were identical with slightly lowered occurrences (around 2–5% difference for each respective IRAEs when contrasted to combined therapy). Encorafenib monotherapy had the lowest adverse effect profile. Most of these IRAEs led to treatment cessation or adjustment, with GI, vision, and ejection fraction disturbances commonly reported.

Overall, the AEs were manageable and improved with time on treatment. In previous COLUMBUS and CoBRIM trials, the toxicity of treatment was equally demonstrated to decrease with time. In summary, the 5-year analysis demonstrates the benefits of 12 months of combined adjuvant therapy (encorafenib plus binimetinib) for unresectable or metastatic BRAF V600 mutated melanoma with long-lasting effects well beyond the treatment timeframe. While the OS for combined therapy and encorafenib monotherapy were both a staggering 35%, the PFS was markedly elevated for combined treatment. 

#### 6.1.4. SECOMBIT

The SECOMBIT trial is a non-comparative randomized three-arm, phase 2 trial evaluating the use of IPI plus NIVO and encorafenib plus binimetinib for untreated BRAF-V600 mutant metastatic melanoma [74]. A total of 209 patients were each randomly assigned to one of the three arms of treatment. Group A received encorafenib (450 mg orally once daily) plus binimetinib (45 mg orally twice daily) until disease progression. They then were administered IPI (3 mg/kg once every 3 weeks) plus NIVO (1 mg/kg once every 3 weeks for four cycles, then 3 mg/kg every 2 weeks). Group B received IPI plus NIVO until disease progression, then encorafenib plus binimetinib combined therapy. Group C was given encorafenib plus binimetinib for 8 weeks, then IPI plus NIVO until disease progression, followed by encorafenib plus binimetinib. To note, the doses for each immunotherapy type were identical between groups.

Results at 2-year follow-up, showed OS rates of 65% in arm A, 73% in arm B, and 69% in arm C. At 3 years, OS rates were 54% (arm A), 62% (arm B), and 60% (arm C). RFS rates at 2 years, compared to 3 years, were 46% then 41% (arm A), 65% then 53% (arm B), and 57% then 54% (arm C). These results show comparable treatment outcomes on OS and RFS, with arm B revealing better outcomes in OS and RFS. However, this contrasts with the best overall response rates (BORRs). For the first treatment, arm A’s rates were 87% while arm B’s were a mere 44.9%, and arm C’s were 82.4%. Nevertheless, in the second treatment, the BORRs were 25.7%, 57.9%, and 62.2%, respectively.

IRAEs of grade 3 or higher were reported in 39% of group A, 59% of group B, and 38% of group C. These commonly included creatine kinase, transaminase, and lipase increases. Discontinuation occurred in 10%, 10%, and 9%, respectively, in arms A, B, and C. No deaths or discontinuation of therapy due to IRAEs were reported.

Therefore, the SECOMBIT trial demonstrates that sequential immunotherapy can significantly impact and increase the survival benefits for patients with treatment-naïve BRAF^V600^-mutated melanoma.

#### 6.1.5. The DREAMseq Trial-ECO-ACRIN EA6134

The DREAMseq Trial-ECOG-ACRIN EA6134 is a phase 3 trial investigating the use of a combination of Dabrafenib plus Trametinib versus a combination of NIVO plus IPI for patients with advanced treatment-naïve BRAF^V600^ mutated melanoma [75]. A total of 265 patients were randomly assigned to receive either NIVO plus IPI (arm A) or dab plus tram (arm B) in the first step. Once the disease progressed, the patients were to be switched to the other therapy, dabr plus tram became arm C, and NIVO plus IPI arm D. The doses were as follows: arm A was given NIVO (1 mg/kg every 3 weeks for four doses then 240 mg IV every 2 weeks for 72 weeks) plus IPI (3 mg/kg every 3 weeks for four doses) and arm B: dab (150 mg twice daily, orally) plus tram (2 mg once daily, orally). The step 2 doses were identical to those of step 1.

At 2-year follow-up, the OS in patients who began with arm A was 71.8% while for arm B was 51.5%. The 3-year OS rates equally demonstrated better results for those beginning with arm A. Median RFS equally favored initiation with arm A with a *p*-value of 0.054. It is important to specify that arm B had over double the amount of BRAF^V600K^ mutated tumors compared to arm A (25.2% vs. 12.1%).

Grade 3 and above IRAEs were present in 59.5% of patients in arm A, 53.1% in arm B, 53.8% in arm C, and 50% in arm D. NIVO plus IPI treatment were more likely to cause fever and leukopenia while dab plus tram was more likely to cause hyponatremia. Initially, 18% of patients on arm A died within 10 months of starting therapy; it is crucial to note that their disease was more aggressive, and their therapy was lower than the rest of the study participants. This, however, does not imply that patients with severe disease should begin with arm B but rather that if they are not responding to step 1, the threshold for switching to step 2 should be lowered to ensure better survival. Additionally, the durability of response in those beginning with arm A is a finding that confirms those of the SECOMBIT trial. 

Thus, the data from the DREAMseq trial suggest improved outcomes with the use of combined IPI plus NIVO therapy followed by BRAF/MEK-targeted therapy (Dabrafenib plus Trametinib).

### 6.2. IO Therapy for Metastatic Melanoma

#### 6.2.1. Long-Term Survival of the CheckMate 067 Trial

This phase 3 double-blind trial reports on the use of combined NIVO plus IPI therapy [76]. However, here, OS is measured after a 7.5-year follow-up, providing longer-term results. Patients had stage III unresectable or stage IV melanoma with confirmed BRAF^V600^ mutation. Three regimens were administered with patients divided based on the presence or absence of BRAF mutation, PD-L1 status, and presence of metastasis; one group received NIVO 1 mg/kg and IPI 3 mg/kg (NIVO plus IPI) every 3 weeks for four total doses, then NIVO 3 mg/kg every 2 weeks. The other two groups were given NIVO 3 mg/kg every 2 weeks up to 2 years or IPI 3 mg/kg every 3 weeks for four doses, respectively.

In the updated follow-up (7.5 years), the objective response rate in the NIVO plus IPI arm was 50%, the NIVO arm was 42%, and the IPI arm was 14%. Complete response rate to treatment was higher in the NIVO plus IPI group (19%) compared to NIVO (17%) and IPI (2%) [77]. Median recurrence-free survival (PFS) was 11.5 months in the NIVO plus IPI group, 6.9 months in the NIVO group, and 2.9 months in the IPI group. The rate of OS at 7.5-year follow-up for the NIVO plus IPI group was 48%, 42% for the NIVO only, and 22% for the IPI group. A distinction between patients with and without BRAF mutation was noted. The patients without BRAF mutations (BRAF wild type) reached the median OS at 39.1 months for the IPI-NIVO arm, 34.4 months for the NIVO arm, and 18.5 months for the IPI arm. The median OS for patients with BRAF mutation was not reached for the combination (NOVI-IPI) arm, 45.5 months in patients treated with NIVO and 24.6 months for IPI arm. Regarding PD-L1 expressing tumors, the data paralleled the non-BRAF mutated patients, with combination therapy being more efficacious. The median OS in NIVO-IPI, NIVO, and IPI arms were 72.1, 36.9, and 19.9 months, respectively. 

IRAE of grade 3 or 4 occurred in 59% of NIVO plus IPI patients, 21% in NIVO and 28% in IPI only (most commonly gastrointestinal adverse effects). It can be concluded that NIVO plus IPI and NIVO treatments achieved higher success rates than IPI treated patients. 

#### 6.2.2. Three-Year Survival of the CheckMate 511 Trial

This phase 3B/4 double-blind trial reports on the use of combined NIVO plus IPI therapy for stage III and IV untreated, unresectable Melanoma [78]. An amount of 360 patients were randomly divided into two groups, with one receiving treatment with a 1:1 preparation of 1 mg/kg NIVO with 3 mg/kg IPI (NIVO1 plus IPI3) and the other receiving 3 mg/kg NIVO with 1 mg/kg IPI (NIVO3 plus IPI1). All patients received four doses separated by 3 weeks, followed by 480 mg NIVO administered 6 weeks later every 4 weeks until the progression of disease or TRAEs became intolerable. The goal of the study was to test the safety and efficacy of both combination drug regimens in this patient population.

In the 3-year updated follow-up, median overall and progression-free survival were similar between groups. Specifically, the OS and PFS were 61% and 43% for NIVO1 plus IPI3 and 59% and 38% for NIVO3 plus IPI1, respectively [79]. However, treatment was terminated due to TRAEs in 39% and 26% of patients treated with NIVO1 plus IPI3 and NIVO3 plus IPI1, respectively, while maintenance treatment was continued in 42% and 57% of the patients treated with NIVO1 plus IPI3 and NIVO3 plus IPI1, respectively. High-grade TRAEs were significantly more frequent in NIVO1 plus IPI3 (48.3%) vs. NIVO3 plus IPI1 (33.9%). Due to similar efficacy and improved tolerance to treatment, NIVO3 plus IPI1 is the preferred option when compared to NIVO1 plus IPI3. Diarrhea, fatigue, pruritis, and rash were the most frequent TRAEs observed in both groups.

#### 6.2.3. RELATIVITY-047

This phase 3 double-blind trial reports on the outcomes of combined dose therapy of relatlimab (anti-lymphocyte-activating gene 3) and NIVO in untreated metastatic or unresectable melanoma as compared to NIVO alone. A fixed-dose preparation of 160 mg relatlimab and 480 mg NIVO was administered every 4 weeks, with the control receiving only 480 mg NIVO in the same timeframe [80]. At a median follow-up of 19.3 months, while no statistically significant conclusion was reached regarding median OS, median PFS was improved with the use of both immune checkpoint inhibitors (10.2 months) as compared to NIVO alone (4.6 months) [80]. This study highlights the validity of LAG-3 as an additional target in advanced melanoma as compared to PD-1-targeted monotherapy. Additionally, after a median 5.6 treatment duration with relatlimab and NIVO, the safety profile was considered favorable with no new safety signals [81]. However, high-grade TRAEs occurred more frequently in the combined therapy (21.1%) as compared to NIVO alone (11.1%). 

#### 6.2.4. REGN3767 Plus Cemiplimab (NCT03005782)-Ongoing

The combination treatment of fianlimab (REGN3767) and cemiplimab (anti-LAG-3 and anti-PD-1, respectively) showed promising results in patients with advanced melanoma in a phase 1 clinical trial. The overall response rate was 61.2%, and estimated median progression-free survival was 15.3 months. Similar results were observed in patients who had prior treatment. Interestingly, while patients were anti–PD-L1 treatment-naive, many had received prior systemic therapy for melanoma in the adjuvant or neoadjuvant setting (23.5%), including some who had prior anti-PD-1 therapy (13.3%) [82].

The median follow-up was 12.6 months, and the median duration of treatment was 32.9 weeks. High-grade TRAEs, IRAEs, and serious adverse events occurred in a significant portion of patients (43.9%, 65.3%, and 32.7%, respectively), with 16.3% discontinuing treatment as a result. However, rates of IRAEs expected with PD-1 monotherapy were similar to prior studies, with the exception of increased rates of adrenal insufficiency (12.2%) [83].

The study suggests that the combination of fianlimab and cemiplimab is associated with high clinical activity in advanced melanoma patients, comparable to other approved combinations of immune checkpoint inhibitors. Notably, this is the first evidence that dual LAG-3 blockade can yield significant activity in patients with advanced melanoma who have previously received adjuvant anti-PD-1 therapy. A phase 3 trial of this combination in treatment-naïve advanced melanoma patients is ongoing (*NCT03005782*).

### 6.3. TKI and IO Triplet Therapy for BRAF-Positive Metastatic Melanoma

#### 6.3.1. IMspire150

This phase 3 is a randomized, double-blind, placebo-controlled study that aims to test the combination of BRAF, MEK, and immune checkpoint inhibition (triplet therapy) in stage IIIC/IV unresectable BRAF^V600^-mutated melanoma [84]. A total of 514 patients received either atezolizumab (anti-PD-L1) with vemurafenib (BRAF inhibitor) and cobimetinib (MEK inhibitor) or vemurafenib and cobimetinib with an atezolizumab placebo (control group). A dose of 850 mg atezolizumab or placebo control was administered IV on days 1 and 15, vemurafenib was taken orally twice per day, and cobimetinib was taken orally once daily for 21 days on and 7 days off. Treatment was given in a 28-day cycle, with atezolizumab or the placebo being added in the second cycle. LDH levels were controlled for in the patient randomization. 

At the time of the first follow-up (median 18.9 months), PFS was significantly longer in the atezolizumab group at 15.1 months compared to 10.6 months for the control [81]. TRAEs resulted in the conclusion of treatment for 13% and 16% of the atezolizumab and control groups, respectively. At approximately 5 years following the initiation of the trial (the data cutoff time), death occurred in nearly half of the patients in both groups (126 vs. 147 in the atezolizumab and control groups, respectively) [85]. The median OS was 39 months for the atezolizumab group, with a median follow-up of 29.1 months, compared to a median OS of 25.8 months for the placebo control with a median follow-up of 22.8 months. Serious adverse TRAEs were reported in 48% of the atezolizumab group compared to 42% of the control. 

Therefore, while the study demonstrated treatment safety and improved PFS with the triplet therapy in BRAF^V600^-mutant advanced melanoma, there was no improvement in OS in additional follow-up. 

#### 6.3.2. STARBOARD-Safety Lead-In

STARBOARD is an ongoing double-blind, randomized, and placebo-controlled phase 3 clinical trial for triplet therapy in unresectable BRAF^V600^-mutant advanced melanoma. The combination drug regimen being administered is the BRAF, MEK, and immune checkpoint inhibitors encorafenib (enco), binimetinib (bini), and pembrolizumab (pembro), respectively [84]. An amount of 20 patients received 450 mg enco once daily, 45 mg bini twice daily, and 200 mg pembro IV every 3 weeks (COMBO450 plus P), while 17 patients received 300 mg enco once daily, 45 mg bini twice daily, and 200 mg pembro IV every 3 weeks (COMBO300 plus P). The purpose of this trial is to determine optimal dosing, while the primary endpoint was defined as dose-limiting toxicity. A recently published safety lead-in found that COMBO450 plus P was preferable, given similar safety profiles between regimens and the published safety data for each drug [85].

#### 6.3.3. KEYNOTE-022–Part 3

KEYNOTE-022, a PHASE I/II double-blind, randomized phase 2 clinical trial, investigated the safety and efficacy of pembro in combination with dab and tram compared to placebo with dab and tram in patients with previously untreated advanced melanoma [86]. Parts 1–3 of this 5-part study treat stage III/IV BRAF^V600E/K^-mutated advanced melanoma, with the primary endpoint defined as PFS.

The triplet regimen demonstrated higher PFS rates at 24 months (41.0% vs. 16.3%) and longer median duration of response (25.1 months vs. 12.1 months) [87]. At 36.6 months of follow-up, results continued to show significantly improved median PFS (16.9 months) with the triplet therapy compared to the control (10.7 months). OS was also higher for the triplet therapy (63 vs. 51.7%). TRAEs were more common with the triplet regimen, including grade 3–5 (58% vs. 25%), immune-mediated adverse events (52% vs. 15%), and serious TRAEs (40% vs. 23%). Notably, one patient in the triplet arm died from treatment-related pneumonitis [88].

The authors suggested a substantial advantage of triplet therapy across all patient subgroups, particularly in patients younger than 65 years, male patients, and those with elevated lactate dehydrogenase levels at baseline [84]. Therefore, the triplet combination of pembro, dab, and tram results in better outcomes for patients with BRAF^V600E/K^-mutated advanced melanoma, although patients suffered a higher incidence of adverse events compared to the control.

A summary of the past and ongoing clinical trials IO and TKI therapy for metastatic melanoma patients are listed in Table 3.

## 7. Treatment of Melanoma with Brain Metastasis

### 7.1. TKI Therapy for Melanoma with Brain Metastasis

#### COMBI-MB

While previous studies have demonstrated the benefits of dab plus tram treatment for BRAF^V600^-mutant metastatic melanoma, the COMBI-MB trial aims to determine whether this benefit extends to a similar cohort with metastatic brain metastases (MBM). COMBI-MB was a phase 2 multi-cohort clinical trial that included four patient cohorts with varying characteristics. Namely, groups A–D consisted of *BRAF*^V600E^-mutant asymptomatic MBM (with no prior local brain therapy), *BRAF*^V600E^-mutant asymptomatic MBM (with prior local brain therapy), *BRAF*^V600D/K/R^-mutant asymptomatic MBM (with or without prior local brain therapy), and *BRAF*^V600D/E/K/R^ symptomatic MBM (with or without prior local brain therapy), respectively [89]. The primary endpoint of the study was defined as intracranial response rate (IRR) in group A, which was met with 58% of patients showing a response. Cohorts B–D, although of smaller sample sizes, also exhibited IRR (56%, 44%, and 59%, respectively). 

The combination therapy showed manageable safety profiles consistent with previous studies. Despite the promising initial response rates, the durability of responses and progression-free survival was relatively short compared to previous trials in patients without MBM. Dutriaux et al. (2022) reported similar findings in a larger open-cohort, non-randomized phase 3B study [90]. This analysis highlighted the prognostic factors reducing PFS, such as disability, elevated LDH, at least three sites of metastases, and non-naive status.

However, these findings highlight the need for additional research to improve outcomes in patients with MBM and suggest that multidisciplinary combination strategies incorporating dab plus tram may be beneficial. The non-randomized design and small sample sizes in some cohorts limit the extent of interpretation of results for these subsets. Further studies are needed to assess the full impact of the combination therapy on overall survival in patients with MBM. 

### 7.2. IO Therapy for Melanoma with Brain Metastasis

#### 7.2.1. ABC

ABC is an open-label phase 2 clinical trial with three cohorts of 76 total patients with MBM [91]. Patients with asymptomatic brain metastases with no prior local brain therapy were treated with either NIVO1 plus IPI3 4 times every 3 weeks and then NIVO3 every 2 weeks (cohort A) or NIVO3 every 2 weeks only (cohort B). Patients who failed prior local brain therapy, were symptomatic, or had leptomeningeal disease were treated with NIVO3 every 2 weeks (cohort C). The primary endpoint was the best IRR after 12 weeks. 

Preliminary results were published, with a median follow-up of 54 months, describing an OS of 51%, 34%, and 13% for cohorts A–C, respectively [92]. High-grade TRAEs were significantly higher in the dual treatment group (cohort A) at 63% compared to 20% and 13% for cohorts B and C, respectively. Therefore, treatment with NIVO alone or in combination with IPI resulted in safe and effective outcomes that warrant further investigation. 

#### 7.2.2. CHECKMATE 204

CHECKMATE is an open-label phase 2 clinical trial with 94 total asymptomatic patients with MBM and no prior radiation therapy [93]. Patients were treated with either NIVO1 plus IPI3 4 times every 3 weeks and then NIVO3 every 2 weeks or NIVO3 every 2 weeks only. The primary endpoint was IRR defined by stable or responsive disease after 6 months. 

With a median follow-up of 14 months, the IRR and extracranial response rates were 57% and 56%, respectively [93]. Specifically, the rates of partial response, complete response, and stable disease after 6 months were 30%, 26%, and 2%, respectively. High-grade TRAEs were noted in 55% of patients. Similar to ABC, the results of this study indicate meaningful safety and efficacy in the treatment of MBM. 

#### 7.2.3. III NIBIT-M2 

The NIBIT-M2 study evaluated the efficacy and quality of life outcomes of melanoma patients with asymptomatic brain metastases treated with different regimens. Patients were randomized to receive fotemustine (alkylating agent) alone, IPI with fotemustine, or IPI with NIVO. The primary endpoint was OS, and secondary endpoints included PFS, response rates, and health-related quality of life (HRQoL) [94].

At the 7-year follow-up, the combination of ipilimumab plus nivolumab demonstrated significantly improved OS compared to fotemustine alone (42.8% vs. 10.9%). Intracranial response rates and PFS were also notably improved with NIVO plus IPI. HRQoL assessments showed that treatment with NIVO plus IPI did not significantly impair the quality of life of treated patients. In fact, patients receiving this combination experienced less deterioration in HRQoL compared to those receiving other treatments [94].

The long-term efficacy of ipilimumab plus nivolumab suggests that this combination could be considered the standard of care for melanoma patients with asymptomatic brain metastases. Additionally, the absence of long-term IRAEs further supports the safety and efficacy of this regimen, similar to known published safety data for combination immunotherapy. Overall, the study indicates that a meaningful percentage of these hard-to-treat patients may achieve durable responses with NIVO plus IPI.

A summary of the past and ongoing clinical trials of IO and TKI therapy for patients with brain metastases in melanoma are listed in Table 4.

## 8. Discussion

Malignant melanoma remains one of the most dangerous malignancies due to its rapid spread and early development of metastases, which can spread to various sites in the body. The survival rates are significantly influenced by the tumor stage at diagnosis, with localized melanoma exhibiting an impressive 5-year survival rate of nearly 98.3%. In contrast, current data suggests that the 5-year overall survival for metastatic patients undergoing treatment with ICI ranges from 30% to 50%, as indicated by phase III clinical trials and real-world published experiences. Additionally, it continues to be a leading cause of skin cancer-related mortality [1,2,3,4,5,8,9].

Despite extensive efforts, conventional treatments like chemotherapy and radiotherapy have failed to substantially enhance clinical outcomes, primarily due to melanomas’ innate resistance. Consequently, recent years have witnessed a shift toward the development of targeted therapies driven by a deeper understanding of cutaneous tumor pathogenesis. Novel approaches such as immunotherapy and tyrosine kinase inhibitors have garnered significant attention for their potential to augment clinical responses in melanoma patients [8,9,91].

Immune checkpoint inhibitors act by targeting the malfunctioning immune system, stimulating the CD8-positive T cells to destroy cancer cells. These inhibitors, particularly anti-CTLA-4 and anti-PD-1 inhibitors, have transformed the management of various cancers, notably advanced melanoma. They enable tumor regression and long-term control in nearly half of patients, a significant improvement compared to historical rates of 9–11%.

These advancements have revolutionized treatment efficacy across all disease stages, from adjuvant and neoadjuvant settings to unresectable (locally advanced) or metastatic stages. Notably, neoadjuvant and adjuvant therapies hold promise for inducing complete and enduring remission.

As an example, the combination therapy of immune checkpoint inhibitors, particularly the administration of both anti-CTLA-4 and anti-PD-1 agents (ipilimumab and nivolumab), yields the highest 5-year overall survival rates in advanced melanoma. Moreover, it demonstrates significant efficacy in treating melanoma brain metastases compared to single-agent therapies like pembrolizumab. However, combined checkpoint inhibition is associated with more frequent and severe immune-related adverse events, necessitating careful monitoring [4,8,9,12,13,14,77].

For patients diagnosed with BRAF-mutated metastatic melanoma exhibiting extensive and symptomatic disease, the preferred initial treatment approach is to initiate targeted therapy utilizing BRAF and MEK inhibitors. This preference is attributed to the rapid response and high response rates observed with targeted therapy in comparison to ICI therapy. Conversely, for patients with low-volume metastatic disease or asymptomatic patients, the preferred initial treatment is to start with ICI therapy. This preference is supported by findings from trials like the DREAMseq Trial [75], which have demonstrated the achievement of long-term survival outcomes with ICI therapy in such patient populations.

Treating NRAS-mutant melanoma poses considerable challenges, particularly for patients who do not respond to immunotherapy. Studies indicate that NRAS-mutant melanoma may exhibit resistance to immune checkpoint inhibitors (ICIs), potentially stemming from changes in cell surface proteins crucial for T-cell response, akin to TP53-mutant tumors. Currently, clinical trials are exploring the efficacy of MEK inhibitors, either alone or in combination with pan-RAF inhibitors, CDK4/6 inhibitors, or focal adhesion kinase inhibitors, for the management of metastatic NRAS-mutant melanoma [95,96,97].

Recent studies suggest that adding an anti-LAG3 antibody to nivolumab improves PFS with less reported severe immune-related adverse events, although its impact on overall survival and its comparative effectiveness with combined anti-CTLA-4 and anti-PD-1 therapy remain unclear [80].

In addition to the therapies discussed earlier, there is a pressing need for future trials aimed at refining treatments to enhance clinical outcomes across all disease stages, encompassing neoadjuvant, adjuvant, and metastatic settings. These trials may investigate various interventions, including surgery, radiotherapy, targeted therapy, and checkpoint immunotherapy. Similarly, there is a critical need for further research focused on brain metastasis. Future trials should aim to explore and optimize treatments tailored to improve outcomes specifically for patients with melanoma-related brain metastasis.

## 9. Conclusions

Immunotherapy and targeted therapy have transformed the landscape of melanoma treatment, yielding remarkable outcomes across all disease stages, from neoadjuvant to adjuvant and metastatic settings. Particularly noteworthy is the significant progress made in treating brain metastasis, marking a pivotal advancement in melanoma care. Ongoing trials hold promise in elucidating the safety and efficacy of additional immunotherapeutic and targeted therapy options for patients at various disease stages of melanoma. Essential questions persist regarding predictive biomarkers, optimal combination therapies, and the necessity for targeted therapy and immunotherapy. Addressing these queries requires further investigation via rigorous studies aiming to advance precision medicine in the realm of melanoma treatment.

## Figures and Tables

**Table 1:** Summary of the past and ongoing clinical trials for neo-adjuvant therapy administered to melanoma patients.

Name	Phase	Stage	Treatment	Primary Endpoint	Reference
***Past Clinical Trials***					
NCT03698019	II	resectable stage III or IV	pemb neoadjuvant and then adj or directly neo-adj	EFS	[50]
OpACIN and OpACIN-neo	II	high-risk stage III	all received nivo plus ipi but in different dosages and periods	PR, RFS, and OS	[51]
PRADO	II	IIIb–d	nivo plus rela	PR	[52]
NCT02519322	II	resectable stage III	nivo alone or in combination with ipi or rela	PR	[53]
***Ongoing Clinical Trials***					
*NADINA*	III	resectable stage III	for non-BRAF mutant ipi plus nivo for 4 C then 12 C of nivo. For BRAF mutant 2 C of ipi plus nivo then dab plus tram.	EFS	[54]

Abbreviations: pathological response, PR; RFS, recurrence-free survival; OS, overall survival; RBAF, v-raf murine sarcoma viral oncogene homolog B1; IPI, ipilimumab; NIVO, nivolumab; rela, relatlimab; pembrolizumab, prmbro; C, cycle; dabrafenib, dab; trametinib, tram; event-free survival, EFS, adjuvant, adjuvant; neo-adjuvant, neo-adj.

**Table 2:** Summary of the past and ongoing clinical trials for adjuvant therapy administered to melanoma patients.

Name	Phase	Stage	Treatment	Primary Endpoint	Reference
***Past Clinical Trials***					
KEYNOTE-716	II	IIB/IIC	pemb vs. placebo	RFS	[55]
*CheckMate 76K*	III	IIB/IIC	nivo vs. placebo	RFS, DMFS	[56]
*Keynote 053*	III	III, resectable IV	pemb vs. interferon alfa-2b vs. ipi	RFS, OS	[57]
*EORTC 18071*	III	resectable stage III	ipi vs. placebo	RFS	[58]
*CheckMate 915*	III	III B-D, resectable stage IV	ipi and nivo vs. nivo	DFS, PD-L1 ≥ 1%	[59,60]
*KEYNOTE-054*	III	IIIA/B/C	pemb vs. placebo	RFS, PD-L1 ≥ 1%	[61]
CheckMate 238	III	IIB/C, resectable stage IV	ipi vs. nivo	RFS	[62,63]
*KEYNOTE-942*	II	stages IIB/C/D and IV	mRNA-4157-P201 alone or plus pemb	RFS, DMFS	[64]
COMBI-AD	III	III	dab plus tram vs. placebo	RFS	[65]
***Ongoing Clinical Trials***					
*COLUMBUS-AD*	III	IIB/C (BRAF mutant)	enco plus bini vs. placebo	RFS, DMFS, OS	[66]
*KEYVIBE-010*	III	IIIA/B/C	pemb alone or in combination with vibostolimab	RFS, DMFS	[67]

Abbreviations: RFS, recurrence-free survival; OS, overall survival; RBAF, v-raf murine sarcoma viral oncogene homolog B1; ipi, ipilimumab; nivo, nivolumab; pembrolizumab, pemb; dabrafenib, dab; trametinib, tram; versus, vs.; distant metastasis-free survival, DMFS; PD-L1, programmed death-ligand 1; enco, encorafinib; bini, binimetinib.

**Table 3:** Summary of the past and ongoing clinical trials of IO and TKI therapy for metastatic melanoma patients.

Name	Phase	Stage	Treatment	Primary and Secondary Endpoints	Reference
***Past Clinical Trials***					
COMBI-d and COMBI-v	III	IV (BRAF mutant)	Daba plus tram	PFS, OS	[70]
*coBRIM*	III	IV (BRAF mutant)	Cobi plus vemu	PFS, OS	[71]
*COLUMBUS*	III	IV (BRAF mutant)	Enco plus bini vs. vemu or enco	PFS	[73]
*SECOMBIT*	II	IV (BRAF mutant)	Ipi plus nivo vs. enco bini	OS, PFS, DOR, ORR	[74]
*DREAMseq*	III	IV (BRAF mutant)	Ipi plus nivo vs. dab plus tram	OS, PFS, DOR, ORR	[75]
*CheckMate 067*	III	IV (include BRAF mutant)	Ipi plus nivo vs. nivo or ipi alone	OS, PFS	[76]
*CheckMate 511*	IIIB/IV	III-unresectable, IV (include BRAF mutant)	Ipi plus nivo vs. nivo plus ipi in different dosages	AEs, OS, PFS, ORR	[78]
*RELATIVITY-047*	III	III-unresectable, IV (include BRAF mutant)	nivo alone or in combination with rela	PFS	[80]
*IMspire150*	III	III-unresectable, IV (include BRAF mutant)	atezo with vemu and cobi vs. placebo with vemu and cobi	OS, PFS, DOR, ORR	[84]
*STARBOARD*	III	III-unresectable, IV (include BRAF mutant)	enco plus bini and pembro	OS, PFS, DOR, ORR	[85]
*KEYNOTE-022*	II	III-unresectable, IV (include BRAF mutant)	pembro plus dab and tram vs. placebo plus dab and tram	OS, PFS, DOR, ORR	[86,87,88]
***Ongoing Clinical Trials***					
*NCT03005782*	I	IV (include BRAF mutant)	fianlimab plus cemiplimab	safety	[82]

Abbreviations: PFS, progression-free survival; OS, overall survival; ipi, ipilimumab; nivo, nivolumab; pembrolizumab, pemb; versus, vs.; DMFS, distant metastasis-free survival; enco, encorafinib; bini, binimetinib; TKI, tyrosine kinase inhibitor; IO, immunotherapy; dabrafenib, dab; trametinib, tram; cobimetinib, cobi; vemurafenib, vemu; overall response rate, ORR; duration of response, DOR; adverse events, AEs; rela, relatlimab; atezo, atezolizumab.

**Table 4:** Summary of the past and ongoing clinical trials of IO and TKI therapy for patients with brain metastases in melanoma.

Name	Phase	Stage	Treatment	Primary and Secondary Endpoints	Reference
***Past Clinical Trials***					
*COMBI-MB*	II	IV (BRAF mutant)	dab plus tram	intracranial response	[89]
*ABC*	II	IV (include BRAF mutant)	nivo plus ipi vs. nivo	intracranial response	[92]
*CHECKMATE 204*	II	IV (include BRAF mutant)	nivo plus ipi vs. nivo	intracranial response	[93]
*III NIBIT-M2*	III	IV (include BRAF mutant)	Fotemustine vs. ipi plus fotemustine vs. ipi plus nivo.	intracranial response	[94]

Abbreviations: RBAF, v-raf murine sarcoma viral oncogene homolog B1; ipi, ipilimumab; nivo, nivolumab; versus, vs.; TKI, tyrosine kinase inhibitor; IO, immunotherapy; dabrafenib, dab; trametinib, tram.

## Data Availability

Data are contained within the article or are available from the authors upon reasonable request.
